# Household food insecurity and early childhood development: Systematic review and meta‐analysis

**DOI:** 10.1111/mcn.12967

**Published:** 2020-02-12

**Authors:** Klébya Hellen Dantas de Oliveira, Géssica Mercia de Almeida, Muriel Bauermann Gubert, Amanda Souza Moura, Ana Maria Spaniol, Daphne C. Hernandez, Rafael Pérez‐Escamilla, Gabriela Buccini

**Affiliations:** ^1^ Department of Nutrition University of Brasília Brasília Brazil; ^2^ Department of Research, Cizik School of Nursing University of Texas Health Science Center Houston Texas; ^3^ Department of Social and Behavioral Sciences Yale School Public Health New Haven Connecticut

**Keywords:** early childhood development, high and low–middle income countries, household food insecurity, infant, meta‐analysis, systematic review, toddlers

## Abstract

Household food insecurity (HFI) is a powerful stressor negatively associated with early childhood development (ECD). However, no comprehensive review has examined the association of HFI and ECD. Therefore, this systematic review and meta‐analysis investigated the association between HFI and ECD domains and subdomains in children under 5 years old. Peer‐reviewed and grey literature were systematically searched in electronic databases with no year or language restrictions. Studies were eligible if they assessed the association between HFI and one or more ECD domains. Data were extracted using a standard predefined protocol. Meta‐analysis was performed, and the heterogeneity across studies was explored. Nineteen studies were included in the systematic review and 14 in the meta‐analysis. Of the studies, 15 were from high income countries (HICs) and four from low–middle income countries (LMICs). For developmental risk and the cognitive/math and cognitive/school readiness and reading subdomains, the only studies available were conducted in HICs. The meta‐analysis showed that HFI was associated with developmental risk (OR 1.28; 95% CI [1.14, 1.45]), cognitive/vocabulary (OR 0.94; 95% CI [0.90, 0.98]), and cognitive/math (OR 0.84; 95% CI [0.73, 0.96]). HFI was marginally associated with cognitive/school readiness and reading (OR 0.91; 95% CI [0.82, 1.00]) and motor development (OR; 0.91, 95% CI [0.80, 1.04]). HFI was associated with poor ECD in children under 5 years old. Specifically, HFI was associated with developmental risk and poor math skills in studies conducted in HICs and with poor vocabulary skills in studies conducted in both HICs and LMICs. Prospective studies examining HFI and ECD are needed in LMICs.

**Key messages**

- Household food insecurity (HFI) is associated with poor early childhood development (ECD) in children under 5 years old.
- HFI is associated with developmental risk and poor math skills in high income countries (HICs) and with poor vocabulary skills in both HICs and low–middle income countries (LMICs).
- Standardized methods to assess ECD and clear definitions of ECD domains are necessary to allow global‐level comparability.

## 1 INTRODUCTION

Adverse childhood experiences, such as poverty, food insecurity, family stress, neglect, and racial disparities, are risk factors for suboptimal early childhood development (ECD) (Knowles, Rabinowich, Ettinger de Cuba, Cutts, & Chilton, 2016). Specifically, studies have found that household food insecurity (HFI) is a powerful stressor with important implications for cognitive—including vocabulary, memory, attention, problem‐solving, analytical, and academic skills (Hobbs & King, 2018; Johnson & Markowitz, 2018); language—including comprehensive and expressive verbal communication (Saha et al., 2010); motor—including control and coordination of gross and fine movements (Hernandez & Jacknowitz, 2009; Milner, Fiorella, Mattah, Bukusi, & Fernald, 2018); socio‐emotional—including the abilities to regulate emotional responses, behavioural problems, and social interactions (Gill, Koleilat, & Whaley, 2018; Hobbs & King, 2018; Milner et al., 2018; Whitaker, Phillips, & Orzol, 2006); and developmental risk—including poor performance in non‐specific developmental domains (Black et al., 2012; Rose‐Jacobs et al., 2008).

HFI is defined as the lack of physical, social, and economic access to sufficient, safe, and nutritious food to meet the dietary needs and food preferences for an active and healthy life (Food and Agriculture Organization, 2013). HFI can be experienced at different levels of severity (mild, moderate, or severe), and more severe and longer exposure to HFI leads to higher odds that HFI will negatively affect ECD outcomes (Jyoti, Frongillo, & Jones, 2005).

Previous reviews have reported the association of HFI with poor ECD (Cook & Frank, 2008; Pérez‐Escamilla & Vianna, 2012; Shankar, Chung, & Frank, 2017). Cook and Frank (2008) showed that HFI was associated with developmental risk and negative effects on physical function, academic performance, and impaired social skill development in the United States (US) preschool‐aged and school‐aged children. Pérez‐Escamilla and Vianna (2012) described that in the US, HFI was associated with poor performance in psycho‐emotional, social, and academic indicators, beginning in the early years and through adolescence. Shankar et al. (2017) found in high income countries (HICs) that even marginal HFI was associated with adverse ECD outcomes, including developmental risk and impaired cognitive outcomes in infants and toddlers, as well as with externalizing and internalizing behaviours among preschoolers, school‐aged children, and adolescents.

These previous reviews were restricted to HICs, and the results focused on long‐term outcomes throughout childhood and adolescence (Cook & Frank, 2008; Pérez‐Escamilla & Vianna, 2012; Shankar et al., 2017). These limitations called for conducting a comprehensive review to capture the entire body of evidence in HICs and low–middle income countries (LMICs) as well as to explore the association of HFI and ECD outcomes by ECD domains and subdomains, that is, cognitive (executive function, school readiness, reading, vocabulary, and math), language (communication and language comprehension and expression), motor (fine and gross motor), socio‐emotional (externalizing behaviour and hyperactivity and internalizing behaviour and anxiety) and developmental risk.

Therefore, the aim of this systematic review and meta‐analysis was to investigate the association between HFI and ECD domains and subdomains among children under 5 years old in HICs and LMICs.

## 2 METHODS

The protocol for this systematic review and meta‐analysis was registered on PROSPERO prior to starting the literature search (CRD42018091435).

### 2.1 Eligibility criteria

This systematic review followed the guidance of the Preferred Reporting Items for Systematic Reviews and Meta‐Analysis (PRISMA; Moher, Liberati, Tetzlaff, Altman, & Group, 2009). A literature search protocol was prepared on the basis of clearly defined a priori inclusion and exclusion criteria.

Studies evaluating the association between HFI and ECD in infants and toddlers under 5 years old were included. Studies were excluded if they were (a) qualitative or (b) reviews (systematic or not), letters to the editor, books, conference abstracts, study protocols and (c) studies that did not report the association between HFI and ECD. Studies were eligible if they (a) reported the association (coefficient β,and odds ratio) between ECD and HFI and (b) used an experience‐based food insecurity scale to assess HFI (Pérez‐Escamilla, Gubert, Rogers, & Hromi‐Fiedler, 2017). Measuring ECD is complex and consensus on the use of a single tool globally has not been reached. Because tools designed to measure ECD vary widely and may cover a single or multiple ECD domains (Fernald, Prado, Kariger, & Raikes, 2017), we chose to include all studies that measured any domains or subdomains of ECD, regardless of the tool used.

If two or more eligible studies used the same data, only one paper was included, which was selected either because it reported more thoroughly the data or was the most recent publication.

### 2.2 Exposure

The key exposure was HFI (vs. non‐HFI), assessed using experience‐based food insecurity scales (Pérez‐Escamilla et al., 2017). These scales (Household Food Security Survey Module (HFSSM), Core Food Security Module (CFSM), Household Food Insecurity Access Scale (HFIAS), and Household Food Security Scale (HHFS)) assess HFI based on the experience by the respondents of food deprivation, poor dietary quality, and corresponding coping skills in the household (Pérez‐Escamilla et al., 2017). These scales have been validated and are used in many countries worldwide to measure HFI (Marques, Reichenheim, de Moraes, Antunes, & Salles‐Costa, 2015; Pérez‐Escamilla et al., 2017; Saha et al., 2010).

### 2.3 Outcome

There is no consensus on the definition of early childhood. The World Health Organization defines early childhood as ages 0–8 years (World Health Organization, 2019). The American Academy of Pediatrics divides early childhood into ages 0–1 (infants), 1–3 (toddlers), and 3–5 (preschoolers) (American Academy of Pediatrics, 2019). Recent publications emphasize the importance of ECD among children under 3 years (Black et al., 2017). In this systematic review, the outcome was defined as ECD in children under 5 years old.

The ECD outcome was classified into five domains based on the conceptual definitions reported by Fernald et al. (2017): (a) cognitive (including five subdomains: cognitive scores, executive function, school readiness and reading, vocabulary, and math), (b) socio‐emotional (including four subdomains: externalizing behaviour, externalizing behaviour/hyperactivity, internalizing behaviour, and internalizing behaviour/anxiety), (c) language (single domain including comprehensive and expressive verbal communication skills), (d) motor (single domain including fine and gross motor skills), and (e) developmental risk (developmental concerns in non‐specific developmental domains).

Studies were allocated into the predefined domains and subdomains, following a consensus process followed by two study co‐authors (MBG and GB). First, the definition of each domain and subdomain was recorded, according to the literature. Then, the studies were allocated into the corresponding domains or subdomains, according to the description reported by the authors. The same study could be allocated to more than one subdomain if results for additional subdomains were available. We chose not to separate fine and gross motor skills into subdomains because only one study made this distinction. The definitions of domains, subdomains, and description according to what was reported in each study are presented in Table 1.

**Table 1:** Early childhood development domain definitions and subdomains classification

ECD domain	ECD subdomain	Study identification	ECD outcome	Definition reported by the study	Tool used to assess ECD outcome
Developmental risk Developmental risk was defined when the delay was not specific per domain and the evaluation was performed by a global screening test.		Black et al. (2012)	Developmental risk	Developmental risk was defined as having one or more developmental concerns.	Parents' Evaluation of Developmental Status
		Rose‐Jacobs et al. (2008)	Developmental risk	Children with two or more significant concerns are at developmental risk.	Parents' Evaluation of Developmental Status
		Drennen et al. (2019)	Developmental risk	Children with two or more concerns were classified as at developmental risk.	Parents' Evaluation of Developmental Status
Socio‐emotional^b^ Social–emotional skills, temperament, and personal social/adaptive skills	Externalizing behaviour	Hobbs & King (2018)	Externalizing behaviour problems	Externalizing behaviours directed toward others (e.g., aggressive behaviours, whether the child argued a lot, bullied, was disobedient, or destroyed things).	Child Behavior Checklist (CBCL/1.5–5)
		King (2018)	Externalizing behaviour problems	Externalizing behaviours (e.g., fights, hits others, or disobedience).	Child Behavior Checklist– (CBCL/1.5‐5)
		Whitaker et al. (2006)	Aggressive	Not reported	Child Behavior Checklist (CBCL/1.5–5)
		Nagata, Gomberg, Hagan, Heyman, & Wojcicki (2018)	Oppositional defiant score	Not reported	Child Behavior Checklist (CBCL/1.5–5)
		Gill et al. (2018)	High‐frequency discipline	Not reported	“How many days in a typical week does NAME need to be disciplined for his/her behavior?” item adapted from the Home Observation Measurement of the Environment‐Short Form scale.
		Johnson & Markowitz (2018)	Conduct problems	Conduct problems (e.g., how often child pushes or tantrums).	Items drawn from the Preschool and Kindergarten Behavior Scales, 2nd ed. and the Social Skills Rating Scale
		Finch, Yousafzai, Rasheed, & Obradović (2018)	Externalizing behaviour problems	Not reported	Strengths and Difficulties Questionnaire
	Externalizing behaviour/hyperactivity	Johnson & Markowitz (2018)	Hyperactivity	Hyperactivity (e.g., how well child pays attention, resists distraction, sits still*a*).	Items drawn from the Preschool and Kindergarten Behavior Scales, 2nd ed. and the Social Skills Rating Scale
		Nagata et al. (2018)	Attention deficit/hyperactivity	Not reported	Child Behavior Checklist (CBCL/1.5–5)
		Whitaker et al. (2006)	Inattention/hyperactivity	Not reported	Child Behavior Checklist (CBCL/1.5–5)
	Internalizing behaviour/anxiety	Whitaker et al. (2006)	Anxious/depressed	Not reported	Child Behavior Checklist (CBCL/1.5–5)
		Nagata et al. (2018)	Anxiety	Not reported	Child Behavior Checklist (CBCL/1.5–5)
	Internalizing behaviour	Nagata et al. (2018)	Affective score	Not reported	Child Behavior Checklist (CBCL/1.5–5)
		Nagata, Gomberg, Hagan, Heyman, & Wojcicki (2018)^a^	Pervasive developmental	Pervasive developmental symptoms included mothers reporting that their children avoided eye contact, did not answer when called or when spoken to, showed little affection, had a speech problem, exhibited strange behaviour, or were upset by new situations.	Child Behavior Checklist (CBCL/1.5–5)
		Milner et al. (2018)	Personal social	Personal social reflects emotional responses and social interactions.	Inventory (Ages and Stages Questionnaire: I)
		King (2018)	Internalizing behaviours	Internalizing behaviours (e.g., withdrawn, shy, secretive, or refuses to talk).	Child Behavior Checklist(CBCL/1.5‐5)
		Hobbs & King (2018)	Internalizing behaviours	Internalizing behaviours are negative emotions directed toward self (e.g., whether the child worried, sulked a lot, was shy, or refused to talk).	Child Behavior Checklist (CBCL/1.5–5)
Cognitive^b^ Cognitive skills and abilities for the executive function/self‐regulation/effortful control, early academic skills, and approaches to learning.	Cognitive scores	Hernandez & Jacknowitz (2009)^a^	Cognitive scores	Early cognitive and language ability by examining items about early communication skills, expressive and receptive vocabulary, listening comprehension, and early problem‐solving skills.	Bayley Short Form‐Research Edition (BSF‐R) Mental Scale
	Executive function	Obradovíc, Yousafzai, Finch, & Rasheed (2016)^a^	Executive function	Executive function skills include inhibitory control (ability to suppress a dominant response in favour of a subdominant response), working memory (ability to hold, update, and manipulate information in the mind over short periods of time), and cognitive flexibility (ability to switch flexibly between two different dimensions).	Executive function battery
		Obradovíc, Yousafzai, Finch, & Rasheed (2016)^a^	Performance Intelligence Quotient	Performance intelligence (e.g., fluid reasoning, spatial processing, perceptual organization, and visual–motor integration).	Wechsler Preschool and Primary Scale of Intelligence III
	School readiness and reading	Hobbs & King (2018)	Woodcock–Johnson letter–word identification	Not reported	Woodcock–Johnson test of achievement letter–word identification subtest
		Huang, Potochnick, & Heflin (2018)	School readiness in reading assessment score	Reading assessment (e.g., phonological awareness, letter recognition and sound knowledge, and word recognition).	Peabody Picture Vocabulary Test
		Johnson & Markowitz (2018)	Reading	Reading ability evaluated letter and letter–sound knowledge, print conventions, and expressive and receptive vocabulary skills.	Measure developed specifically for the Early Childhood Longitudinal Study‐Birth Cohort
	Vocabulary	Hobbs & King (2018)	Peabody Picture Vocabulary Test	Children's receptive vocabulary capabilities for standard English and academic readiness.	Peabody Picture Vocabulary Test–Revised
		Obradovíc, Yousafzai, Finch, & Rasheed (2016)	Verbal Intelligence Quotient	Verbal intelligence (e.g., vocabulary, verbal comprehension, and knowledge about the world).	Wechsler Preschool and Primary Scale of Intelligence III
	Math	Huang et al., 2018	School readiness in math assessment score	Math assessment (e.g., number sense, counting, operations, and geometry).	Test of Early Mathematics Ability–3
		Johnson & Markowitz (2018)	Math	Math skills assessed children's number sense, properties, operations, measurement, and geometry and spatial abilities.	Measure developed for the Early Childhood Longitudinal Study‐Birth Cohort
		Mickens et al. (2019)	Early Mathematics Ability	Informal and formal mathematical knowledge. Informal knowledge covers concepts that children learn outside of the context of formal schooling, whereas formal knowledge covers concepts that a child is taught in school.	Test of Early Mathematics Ability (TEMA)
Motor^b^ Control and coordination of gross and fine movements skills		Hernandez & Jacknowitz (2009)	Fine and gross motor	Fine motor skills (e.g., reaching, grasping, and manipulating small objects) and gross motor skills (e.g., sitting, standing, walking, and balance).	Motor skills scale adapted from the Bayley Scales of Infant Development, Second Edition
		Milner et al. (2018)	Gross motor	Gross motor evaluates body and muscle movement, including tasks such as standing, walking, and balancing.	Inventory (Ages and Stages Questionnaire: I)
Language^b^ Comprehensive and expressive verbal communication skills		Milner, Fiorella, Mattah, Bukusi, & Fernald (2018)^a^	Communication	The communication assesses language development and the use of words or sounds to express feelings.	Inventory (Ages and Stages Questionnaire: I)
		Saha et al. (2010)^a^	Language comprehension	Children's ability to comprehend words in different categories (e.g., animals, body parts, or food).	Bengali adaptation of MacArthur's Communicative Development Inventory
		Saha et al. (2010)^a^	Language expression	Children's ability to express words in different categories (e.g., animals, body parts, or food).	Bengali adaptation of MacArthur's Communicative Development Inventory

Abbreviation: ECD, early childhood development.

a Hernandez & Jacknowitz, 2009 (evaluating cognitive scores), Obradovíc et al., 2016 (evaluating executive function and performance intelligence quotient), Nagata et al., 2018 (evaluating pervasive developmental), Milner et al., 2018 (evaluating communication), and Saha et al., 2010 (evaluating language comprehension and language expression) could not be grouped in the ECD domains due to the ECD definition.

b The four main ECD domains were adapted according the proposed by Fernald et al. (2017).

### 2.4 Search strategy

Seven electronic bibliographic databases (Medline/PubMed, LILACS, Scopus, Web of Science, Science Direct, EMBASE, and PsycINFO) were systematically searched. In addition, grey literature was searched for on Google Scholar and Open Grey. The search on Google Scholar was limited to the first 400 records, as recommended by previous authors (Pizato, Botelho, Gonçalves, Dutra, & de Carvalho, 2017). All searches were conducted without language or date restrictions, through April 20, 2018, and the search was updated on November 13, 2019. Reference lists of eligible articles were read to identify additional studies related to the topic. Co‐authors (DCH, MBG, RPE, and GB)) were consulted on the inclusion of additional studies that were not captured by the systematic search. This procedure was used to ensure that relevant studies were not left out of the review.

Articles were identified by using the following terms: infant, toddler, food insecurity, food security, and ECD. Descriptors for these terms were identified in English, Portuguese, and Spanish from the Medical Subject Headings (MeSH) terms (Data S1). The searches were conducted independently by two co‐authors (KHDO and GMA). The studies identified from each search were imported into EndNote X8 (Thomson Reuters 2018) prior to removing duplicates.

### 2.5 Study selection

Articles were excluded if they did not meet the aforementioned criteria (Data S2). Two co‐authors (KHDO and GMA) independently screened the titles and abstracts. Studies were included in the initial screening if they examined the association between HFI and ECD. Next, the articles were independently assessed for eligibility using the predefined inclusion and exclusion criteria defined above. Disagreements were resolved through consensus, or when required, through consultation with a third co‐author (ASM).

### 2.6 Data extraction

Data were extracted independently by two co‐authors (KHDO and GMA). The data extraction form included study identification (author, year of publication, and country of study, country's income level), name of the primary study and year of data collection, study design and sample size, child's age, HFI (status, tool, child's age when measured, and prevalence), ECD (tool, child's age when measured, and outcomes), effect size of the association between ECD and HFI (odds ratio or coefficient β with respective 95% confidence interval—adjusted or crude), and confounders. When necessary, authors were contacted to provide missing information or additional data to calculate the OR and CI of the association between HFI and ECD.

### 2.7 Quality assessment of studies

Quality assessment was conducted by reviewing key study design and methodological attributes based on a checklist adapted from the Effective Public Health Project Practice Quality Assessment Tool checklist (“http://www.ephpp.ca/tools.html”) (Armijo‐Olivo, Stiles, Hagen, Biondo, & Cummings, 2012). This quality assessment tool has been used and adapted by other systematic reviews in the maternal and child nutrition field with reliable and consistent results regarding studies quality assessment (Boccolini, Carvalho, & Oliveira, 2015; Buccini, Pérez‐Escamilla, Paulino, Araujo, & Venancio, 2017).

The following six attributes were classified as either “strong,” “moderate,” or “weak”: (a) selection bias, (b) study design, (c) confounding factors, (d) blinding, (e) data collection methods, and (f) withdrawals and dropouts. The questions to assess the following three attributes—confounding factors, blinding, and withdrawals and dropouts—were adapted to capture the quality of observational studies. Confounding factors assessed if there were or not significant differences between HFI group versus household food security (examples of confounders include race, sex, age, and health status). Blinding assessed if the interviewer assessing the ECD outcome was aware of the HFI status of participants. Withdrawals and dropouts assessed if follow‐up losses (in longitudinal studies) or missing data were reported. A roadmap for scoring the six attributes (a–f) is described in Data S3. Study quality was upgraded or downgraded based on the internal validity of the studies. The studies were classified according to the final score of Effective Public Health Project Practice Quality Assessment Tool as “strong quality” if none attribute was weak; “moderate quality” if one of the six attributes was classified as weak; and “weak quality” for studies with more than one attribute classified as weak (Armijo‐Olivo et al., 2012). The original and adapted checklist questions are presented in Data S3.

### 2.8 Data analysis

#### 2.8.1 Effect size

Effect measures were presented as pooled odds ratios. For studies that summarized the effect size with estimators other than ORs, whenever possible, the estimators were converted to ORs, and the 95% CI was calculated (Deeks, Altman, & Bradburn, 2008). For four studies, the conversion to OR was not possible due to lack of information; therefore, they were excluded from the meta‐analysis (Fuller et al., 2002; Greder, Peng, Doudna, & Sarver, 2017; King, 2018; Zaslow et al., 2009). When adjusted estimates were available, they were included; otherwise, crude estimators were considered (Gill et al., 2018; Obradovíc et al., 2016).

When studies reported two or more ECD outcomes, the findings were included in the meta‐analysis separately, by ECD domain and subdomain. If more than one ECD outcome per domain was presented in the same study, they were grouped according to predefined ECD domains and subdomains definitions. Domain definitions as well as how each study outcome were defined and grouped within subdomains are presented in Table 1.

#### 2.8.2 Meta‐analysis

Effect measures from the meta‐analysis were presented as pooled odds ratios (ORs) by ECD domain (developmental risk and motor) and, when appropriate, by subdomains (cognitive and socio‐emotional). The cognitive domain was represented by three subdomains: cognitive/school readiness and reading, cognitive/math, and cognitive/vocabulary. The socio‐emotional domain was represented by four subdomains: internalizing behaviour, internalizing behaviour/anxiety, externalizing behaviour, and externalizing behaviour/hyperactivity. The language domain was excluded from the meta‐analysis because the two studies identified assessed different aspects of language and could not be combined in a single subdomain.

To interpret meta‐analysis results, high scores for developmental risk and socio‐emotional outcomes (internalizing behaviour, internalizing behaviour/anxiety, externalizing behaviour, and externalizing behaviour/hyperactivity) indicate poor development. On the other hand, low scores for cognition and motor development domains reflect poor development.

Heterogeneity was assessed by the degree of inconsistency (I^2^ statistic) and its significance (*p* < .05), using a fixed effect model. If high heterogeneity was found (i.e., I^2^ > 50%) random‐effects models were used for the analysis (Deeks et al., 2008). Random‐effects models take into account the variability of effects between different studies (Deeks et al., 2008). Meta‐regression was considered for studies with high heterogeneity (i.e., I^2^ > 50%); however, due to the relatively low number of studies, we were unable to follow this approach. Instead, we conducted a descriptive analysis of possible sources of heterogeneity. Results were not broken down by country income due to the scarcity of studies conducted in LMICs. This decision is unlikely to have biased our results because this source of heterogeneity was considered in the interpretation of the findings, and findings when just including HICs were similar as when also including LMICs. All analyses were conducted using Stata version 14.1 (Stata Corp., College Station, TX, USA).

## 3 RESULTS

### 3.1 Characteristics of studies

**Figure 1:**
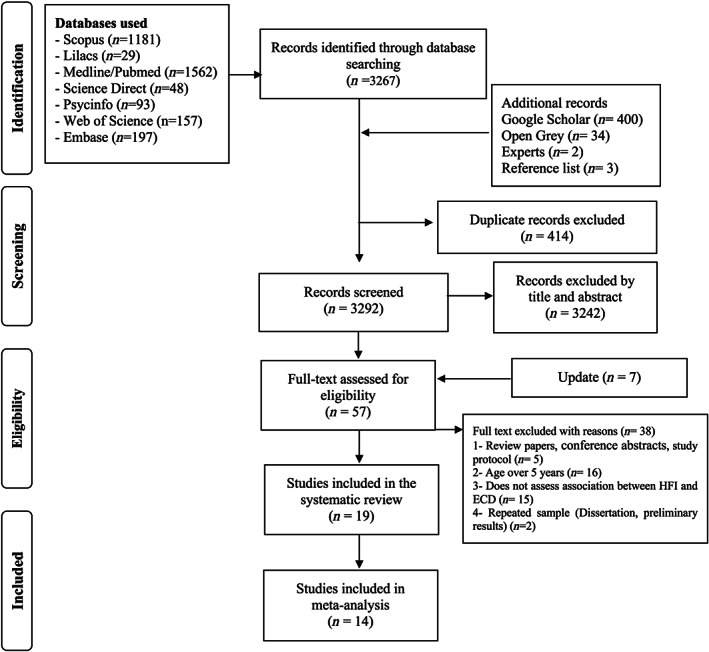
Preferred Reporting Items for Systematic Reviews and Meta‐Analysis flow diagram of systematic review on household food insecurity and early childhood development

**Figure 2:**
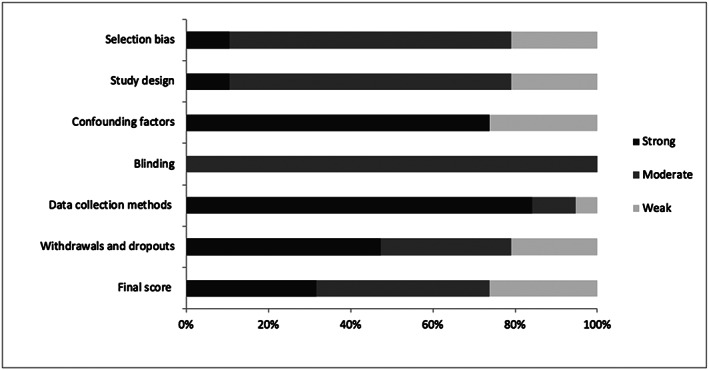
Summary of the risk of bias of the studies included in the systematic review based on the checklist “Effective Public Health Practice Project Quality Assessment Tool”

A total of 3,706 publications were identified in the initial search. After excluding 414 duplicates, a total of 3,292 publications were screened for exclusion criteria by title and abstracts. This resulted in 57 publications for full text reading, including seven from the search update (November 13, 2019). Of those, 38 studies did not meet the eligibility criteria and were excluded. The reasons for exclusion are described in Data S2. Thus, 19 studies were included in this systematic review, and 14 studies were included in the meta‐analysis (Figure 1). In the quality assessment, five studies were classified as weak, eight as moderate and six as strong. Figure 2 indicates that 31.6% of the studies had strong quality. The quality attributes examined that were the weakest were confounding factors followed by selection bias, study design, and withdrawals and dropout.

Table 2 summarizes the main characteristics of the studies included in the systematic review. The systematic review included 15 studies from the US (78.9% of the studies). Only four studies were conducted in LMICs: Kenya (Milner et al., 2018), Pakistan (Finch et al., 2018; Obradovíc et al., 2016), and Bangladesh (Saha et al., 2010). In the majority of studies, HFI and ECD were measured at the same time or in an interval up to 24 months, and six studies did not present this information (Black et al., 2012; Drennen et al., 2019; Gill et al., 2018; Greder et al., 2017; Hobbs & King, 2018; Mickens, 2019). Information regarding HFI and ECD status was usually reported by the mothers. All HFI scales probed for HFI in the context of food deprivation due to lack of money. All scales, but one (HHFS), were directly adapted from the Household Food Security Survey Module(HFSSM).

**Table 2:** General characteristics of the studies that evaluated the association between household food insecurity and early childhood development by domains

Study identification Country's income level^e^	Name of the primary study Year of data collection	Study design Sample size^g^ (*n*)	Quality score	Child's age	HFI		ECD
					Status	Tools Child's age when measured	Tools Child's age when measured
Developmental risk^d^							
Drennen et al. (2019) United States High income	Children's HealthWatch 2009–2017	Cross‐sectional 28,184	Weak	4–48 months	HFI = low and very low FS	18‐item HFSSM N/R	PEDS N/R
Black et al. (2012) United States High income	Children's HealthWatch 2000–2010	Cross‐sectional 26,950	Weak	4–36 months	HFI = low and very low FS	18‐item CFSM N/R	PEDS N/R
Rose‐Jacobs et al. (2008) United States High income	Children's HealthWatch 2004–2005	Cross‐sectional 2,010	Weak	4–36 months	HFI = low and very low FS HFI with hunger	18‐item CFSM 4–36 months	PEDS 4–36 months
Socio‐emotional^d^							
Finch et al. (2018) Pakistan Low–middle income	PEDS trial 2011–2012 to 2013–2014^a^	Longitudinal 1,302	Strong	24 and 48 months	HFI	HFIAS 24 months and 48 months	SDQ 48months. BSID‐III 24months
Gill et al. (2018) United States High income	WIC 2014	Cross‐sectional 4,125	Weak	6 months–5 years	HFI = low and very low FS	6‐item HFSSM N/R	1 question (adapted from the HOMES‐SF scales) N/R
Hobbs & King (2018) United States High income	FFCWS 2003 and 2006 1	Longitudinal^f^ 2,046	Moderate	5 years	HFI = low and very low FS	18‐item HFSSM N/R	CBCL N/R
Johnson & Markowitz (2018) United States High income	ECLS‐B 2001, 2003, and 2005‐2006	Longitudinal 3,700	Strong	9, 24, and 57^b^ months	HFI = low and very low FS	18‐item CFSM 9 months, 2 years, and preschool	BSF‐R. Preschool and Kindergarten Behavior Scales Social Skills Rating Scale 9 months, 2 years, and preschool
King (2018) United States High income	FFCWS 2003 and 2005^a^	Longitudinal 2,044	Strong	3 and 5 years	HFI	18‐item HFSSM 3 and 5 years^c^	CBCL 3 and 5 years^c^
Milner et al. (2018) Kenya Low–middle income	Panel study on fishing livelihoods, fish consumption, and child nutrition 2012–2015	Longitudinal 304	Moderate	<2 years	HFI Timing HFI Intensity HFI Duration HFI	9‐item HFIAS 0–3–6–9–12–15–18–21–24 months	Inventory ASQ:I 6–12–18–24 months
Nagata et al. (2018) United States High income	Hispanic eating and nutrition cohort 2010–2011 to 2012–2013^a^	Longitudinal^f^ 168	Moderate	4 and 5 years	HFI	18‐item HFSSM 4 year	CBCL 5 years
Greder et al. (2017) United States High income	RFSH 2011 and 2012	Longitudinal^f^ 175	Moderate	18–60 months	HFI	6‐item HFSSM N/R	CBCL N/R
Zaslow et al. (2009) United States High income	ECLS‐B 2001–2002 and 2003–2004	Longitudinal^f^ 8,944	Moderate	9 and 24 months	HFI = very low FS	18‐item HFSSM 9 months	TAS‐45 24 months
Whitaker et al. (2006) United States High income	FFCWS 2001–2003^a^	Longitudinal^f^ 2,870	Strong	3 years	HFI = low and very low FS	10‐item HFSSM 3 years	CBCL 3 years
Fuller et al. (2002) United States High income	1998	Longitudinal^f^ 405	Strong	24–42 months	FS and hunger index	12‐item CFSM 9, 24, and 48 months^c^	CBCL (adapted) 9, 24, and 48 months^c^
Cognitive^d^							
Mickens et al. (2019) United States High income	LSCA 2010–2011	Longitudinal 792	Moderate	3–5 years	HFI = low and very low FS	8‐item HFSSM N/R	TEMA N/R
Hobbs & King (2018) United States High income	FFCWS 2003 and 2006 1	Longitudinal^f^ 2,046	Moderate	5 years	HFI = low and very low FS	18‐item HFSSM N/R	PPVT (revised) Woodcock–Johnson test of achievement letter–word identification subtest N/R
Huang et al. (2018) United States High income	ECLS‐B 2005–2006^a^	Longitudinal^f^ 8,900	Moderate	48 months	HFI = low and very low FS	18‐item CFSM 48 months	PPVT Test of Early Mathematics Ability‐3 48 months
Johnson & Markowitz (2018) United States High income	ECLS‐B 2003, and 2005–2006	Longitudinal 3,700	Strong	9, 24, and 57^b^ months	HFI = low and very low FS	18‐item CFSM 9 months, 2 years, and preschool	BSF‐R 9 months, 2 years, and preschool
Obradovíc, Yousafzai, Finch, & Rasheed (2016) Pakistan Low–middle income	PEDS trial 2011–2012 to 2013–2014^a^	Longitudinal^f^ 8,944	Moderate	24–48 months	HFI	HFIAS 24 months	WPPSI‐III inhibitory control task working memory task cognitive flexibility task 48 months
Hernandez & Jacknowitz (2009) United States High income	ECLS‐B 2001 and 2003	Longitudinal 7,900	Strong	9 and 24 months	HFI = low, very low, and marginally FS Persistent adult HFI, Transitional adult HFI	10‐item HFSSM 9 and 24 months	BSF‐R 9 and 24 months
Zaslow et al. (2009) United States High income	ECLS‐B 2001–2002 and 2003–2004	Longitudinal^f^ 8,944	Moderate	9 and 24 months	HFI = very low FS	18‐item HFSSM 9 months	BSF‐R 24 months
Motor^d^							
Milner et al. (2018) Kenya Low–middle income	Panel study on fishing livelihoods, fish consumption, and child nutrition 2012 to 2015	Longitudinal 304	Moderate	<2 years	HFI Timing HFI Intensity HFI Duration HFI	9‐item HFIAS 0–3–6‐9–12–15–18–21–24 months	Inventory ASQ: I 6–12–18–24 months
Hernandez & Jacknowitz (2009) United States High income	ECLS‐B 2001 and 2003	Longitudinal 7,900	Strong	9 and 24 months	HFI = low, very low and marginally FS Persistent adult HFI, Transitional adult HFI	10‐item HFSSM 9 and 24 months	BSF‐R 9 and 24 months
Language^d^							
Milner et al. (2018) Kenya Low–middle income	Panel study on fishing livelihoods, fish consumption, and child nutrition 2012–2015	Longitudinal 304	Moderate	<2 years	HFI Timing HFI Intensity HFI Duration HFI	9‐item HFIAS 0–3–6–9–12–15–18–21–24 months	Inventory ASQ:I 6–12–18–24 months
Saha et al. (2010) Bangladesh Low–middle income	MINIMat 2002 and 2003	Longitudinal^f^ 1,639	Weak	18 months	HFI	11‐item HHFS 8 and 30 weeks of pregnancy	MacArthur's CDI (adaptation) 18 months

Abbreviations: ASQ, Ages and Stages Questionnaire; BSF‐R, Bayley Short Form‐Research Edition; BSID‐III, Bayley Scales of Infant and Toddler Development, Third Edition BSID‐III; CBCL, Child Behavior Checklist; CDI, Communicative Development Inventory; CFSM, Core Food Security Module; ECD, early childhood development; ECLS‐B, Early Childhood Longitudinal Study‐Birth Cohort; FFCWS, The Fragile Families and Child Wellbeing Study; FS, food security; HFI, household food insecurity; HFIAS, Household Food Insecurity Access Scale; HFSSM, Household Food Security Survey Module; HHFS, household food security; HOME‐SF, The Home Observation Measurement of the Environment‐Short Form; LSCA, Longitudinal Child Study of Arizona; MINIMat, Maternal and Infant Nutrition Intervention in Matlab; N/R, not reported; PEDS, Parents Evaluation of Developmental Status; PEDS trial, Pakistan Early Child Development Scale‐Up; PPVT, Peabody Picture Vocabulary test; RFSH, Rural Families Speak about Health; SDQ, Strengths and Difficulties Questionnaire; TAS‐45, Toddler Attachment Sort‐45; TEMA, Test of Early Mathematics Ability; WIC, Nutrition Program for Women, Infants, and Children; WPPSI‐III, Wechsler Preschool and Primary Scale of Intelligence III.

a Year of data collection calculated from date provided by the study.

b Age verified by ECLS website.

c Child's age when HFI and ECD was measured by the study author.

d Reported as ECD description adopted in this review.

e Reported as proposed by The World Bank. Countries and Economies: Income levels, 2019.

f Studies originally designed as longitudinal presenting cross‐sectional analysis for the purposes of this review were classified within study design as longitudinal.

g Sample size for the purposes of this review.

Fifteen studies had longitudinal design, however nine of these studies presented cross‐sectional analysis for the purposes of this review (Fuller et al., 2002; Greder et al., 2017; Hobbs & King, 2018; Huang et al., 2018; Nagata et al., 2018; Obradovíc et al., 2016; Saha et al., 2010; Whitaker et al., 2006; Zaslow et al., 2009). Four studies had cross‐sectional design (Black et al., 2012; Drennen et al., 2019; Gill et al., 2018; Rose‐Jacobs et al., 2008). Confounders used to adjusted the association between HFI and ECD were related to the (a) child characteristics (i.e., gender, age, birth weight, race/ethnicity, nutrition status, and breastfeeding history), (b) caregiver characteristics (i.e., maternal education, maternal employment, maternal race/ethnicity, and marital status), and (c) household characteristics (i.e., public benefits receipt, income, size, and number of children).

### 3.2 Description of HFI and ECD outcomes

The prevalence of HFI in the US among 9‐month‐old infants was 12.5%, according to a nationally representative longitudinal study (Zaslow et al., 2009) and 11.7% at 48 months old (Huang et al., 2018; Table 3). In LMICs, the prevalence of HFI was 33.0% at 24 months and 37.0% at 48 months of age (Obradovíc et al., 2016).

**Table 3:** Main results of studies evaluating the association between household food insecurity and early childhood development by domains

Study identification	ECD outcomes (% delay or x̅)	HFI prevalence	ECD (% delay, x̅ or r) by HFI	Effect size	Effect size (ORs used in the meta‐analysis)
Developmental risk^d^					
Drennen et al. (2019) United States	11.5%	27.3% overall HFI 14.0% as HFI/child secure 13.3% as HFI/child FI	12.5% HFI/Child Secure 14.0% HFI/Child FI	<13 mo HFI/child secure OR_a_ 1.12 (0.85–1.47) HFI/child FI OR_a_ 1.49 (1.11–1.98) 13–24 months HFI/child secure OR_a_ 1.12 (0.90–1.39) HFI/child FI OR_a_ 1.22 (0.98–1.53) 25–36 months HFI/child secure OR_a_ 1.23 (0.98–1.54) HFI/child FI OR_a_ 1.35 (1.08–1.69) 37–48 months HFI/child secure OR_a_ 1.32 (1.02–1.72) HFI/child FI OR_a_ 1.44 (1.12–1.85)	25–36 months HFI/child secure OR_a_ 1.23 (0.98–1.54)
Black et al., (2012) United States	15.2%^a^	24.0%	N/R	HFI OR_a_ 1.22 (1.22–1.69)	HFI OR_a_ 1.22 (1.22–1.69)
Rose‐Jacobs et al. (2008) United States	14.0%	21.0% HFI 6.0% HFI with hunger	18.0%	HFI OR_a_ 1.76 (1.26–2.46) HFI without hunger OR_a_1.77 (1.23–2.56)	HFI OR_a_ 1.76 (1.26–2.46)
Socio‐emotional^d^					
Finch et al., (2018) Pakistan	Externalizing behaviour problems x̅ = 0.951 SD = 0.523 Prosocial behaviours x̅ = 1.529 SD = 0.363 BSID‐III social–emotional x̅ = 93.709 SD = 18.334 BSID‐III cognitive‐language x̅ = 80.5 SD = 12.793	32.8% HFI	N/R	Externalizing behaviour β 0.187 SE (0.068) Prosocial behaviours β −0.008 SE (0.061)	Externalizing behaviour OR_a_ 1.21 (1.06–1.38)^e^
Gill et al., (2018) United States	High discipline frequency 56.4%	22.7% low FS 9.7% very low FS 67.6% marginal/high FS	High discipline frequency Low FS = 60.7% Very low FS = 64.2%	High‐frequency discipline Low FS OR_a_ 1.27 (1.07, 1.49) Very low FS OR_a_ 1.41 (1.11, 1.79)	High‐frequency discipline Low and very low FS OR 1.38 (1.21–1.58)^e^
Hobbs & King (2018) United States	Externalizing behaviours x̅ = 9.6 Internalizing behaviours x̅ = 4.3	19.2% HFI 4.0% very low FS	N/R	Externalizing behaviours 10th β 0.02 (−0.05 to 0.10) 25th β 0.24 (0.12 to 0.36) 50th β 0.27 (0.15 to 0.39) 75th β 0.28 (0.10 to 0.46) 90th β 0.32 (0.08 to 0.57)Internalizing behaviours 10th β 0.28 (0.22 to 0.33) 25th β 0.25 (0.15 to 0.35) 50th β 0.39 (0.26 to 0.52) 75th β 0.43 (0.26 to 0.61) 90th β 0.44 (0.25 to 0.63)	Externalizing behaviours 90th OR_a_ 1.38 (1.08–1.77)^e^ Internalizing behaviours 90th OR_a_ 1.55 (1.28–1.88)^e^
Johnson & Markowitz (2018) United States	Bayley behaviour score at 9 months x̅ = 5.16 SD = 0.99 Bayley behaviour score at 2 years x̅ = 3.41 SD = 0.83	Low FS at 9 months x̅ = 0.15 Very low FS at 9 months x̅ = 0.04 Low FS at 2 years x̅ = 0.11 Very low FS at 2 years x̅ = 0.03 Low FS at preschool x̅ = 0.16 Very low FS at preschool x̅ = 0.05	Bayley behaviour score at 9 months HFI at 9 months x̅ = 5.13 SD = 0.99 HFI at 2 years x̅ = 5.14 SD = 0.98 HFI at preschool x̅ = 5.13 SD = 1.00 Bayley behaviour score (2 years) HFI at 9 months x̅ = 3.41 SD = 0.84 HFI at 2 years x̅ = 3.39 SD = 0.84 HFI at 2 years x̅ = 3.38 SD = 0.83	Hyperactivity Low FS at 2 years β −0.01 SE (0.09) Very low FS at 2 years β 0.41 SE (0.15) Conduct problems Low FS at 2 years β −0.01 SE (0.09) Very low FS at 2 years β 0.41 SE (0.18)	Hyperactivity Low FS at 2 years OR_a_ 0.99 (0.83–1.18)^e^ Conduct problems Low FS at 2 years OR_a_ 0.99 (0.83–1.18)^e^
King (2018) United States	N/R	19.0% HFI 7.0% child HFI	HFI Externalizing behaviours at 5 year x̅ = 11.9 Internalizing behaviours at 5 year x̅ = 5.9 Child HFI Externalizing behaviours at 5 year x̅ = 12.3 Internalizing behaviours at 5 year x̅ = 5.9	Externalizing behaviours Child HFI SD 0.07 CSE (0.02) HFI SD 0.02 CSE (0.01) Internalizing behaviours Child HFI SD 0.06 CSE (0.03) HFI SD = 0.03 CSE (0.01)	Not possible to calculate OR
Milner et al. (2018) Kenya	N/R	HFI timing Round 1 x̅= 0.94 SD = 0.51 Round 2 x̅= 0.88 SD = 0.46 Round 3 x̅= 0.81 SD = 0.44 Round 4 x̅=0.83 SD = 0.40 Round 5 x̅= 0.83 SD = 0.43 Round 6 x̅=0.80 SD = 0.45 Round 7 x̅=0.80 SD = 0.41 Round 8 x̅=0.80 SD = 0.44 Round 9 x̅=0.84 SD = 0.47 HFI intensity Cumulative 24 months x̅=7.54 SD = 2.98 HFI duration Never 35.0% 1–2 times 33.0% 3–4 times 15.0% 5–6 times 9.0% ≥7 times 7.0%	N/R	Personal social HFI 3 months ago β −0.16 (−0.29, −0.031) HFI current β 0.032 (−0.11, 0.18) HFI intensity β −0.021 (−0.054, 0.012) HFI duration β −0.015 (−0.055, 0.026)	Personal social HFI 3 months ago OR_a_ 0.85 (0.75–0.97)^e^
Nagata et al. (2018) United States	6.0% affective score 5.4% anxiety score 5.4% oppositional defiant score 3.6% attention deficit/hyperactivity score 7.1% pervasive developmental problems	8.6% marginal FS 28.2% low FS 4.9% very low FS	N/R	Affective score β 0.13 SE (0.07) Pervasive developmental score β 0.21 SE (0.10) Oppositional defiant score β 0.06 SE (0.08) Anxiety OR_a_ 1.02 (0.81–1.28) Attention deficit/hyperactivity OR_a_ 0.91 (0.65–1.29)	Affective score OR_a_ 1.14 (0.99–1.31)^e^ Oppositional defiant score OR_a_ 1.06 (0.91–1.24)^e^ Anxiety OR_a_ 1.02 (0.81–1.28) Attention deficit/hyperactivity OR_a_ 0.91 (0.65–1.29)
Greder et al. (2017) United States	Internalizing behaviours T score = 50.25, SD = 11.03 CI 29.00–79.00 Externalizing behaviours T score = 51.34, SD = 11.54 CI 28.00–92.00	Score 1.55, SD = 2.04 CI 0.00–6.00	Internalizing child behaviour *r* = .23 Externalizing child behaviour *r* = .35	Externalizing behaviour β 0.236 *p* < .05	Not possible to calculate OR
Zaslow et al. (2009) United States	38.7% insecurely attached	2.6% very low FS 9.9% low FS	N/R	Insecure attachment β 0.013	Not possible to calculate OR
Whitaker et al. (2006) United States	10.4% aggressive 12.6% anxious/depressed 10.2% inattention/hyperactivity 21.9% any behaviour problem	17.1% mothers marginally FS 12.2% mothers HFI 3.5% mothers HFI with hunger. Mothers HFI = 27.4% children HFI Mothers marginally FS = 0.6% children HFI	Aggressive 14.5% marginally FS 20.8% HFI Anxious/depressed 18.9% marginally FS 24.6% HFI Inattention/hyperactivity 13.6% marginally FS 18.6% HFI Any behaviour problem 29.9% marginally FS 38.6% HFI	Aggressive Marginally FS OR_a_ 1.5 (1.1–2.1) HFI OR_a_ 1.9 (1.4–2.7) Anxious/depressed Marginally FS OR_a_ 1.8 (1.4–2.4) HFI OR_a_ 2.2 (1.6–3.1) Inattention/hyperactivity Marginally FS OR_a_ 1.6 (1.1–2.2) HFI OR_a_ 1.9 (1.4–2.8) Any behaviour problem Marginally FS OR_a_ 1.6 (1.3–2.1) HFI OR_a_ 2.1 (1.6–2.7)	Aggressive HFI OR_a_ 1.90 (1.40–2.70) Anxious/depressed HFI OR_a_ 2.20 (1.60–3.10) Inattention/hyperactivity HFI OR_a_ 1.90 (1.40–2.80)
Fuller et al. (2002) United States	Aggression Girls x̅ = 13.8, SD = 8.4 Boys x̅ = 15.4 SD = 8.6 Inattentiveness Girls x̅ = 6.8, SD = 3.9 Boys x̅ = 7.5, SD = 3.4	N/R	N/R	Aggression β 0.07 *p* > .05^c^Inattentiveness β 0.03 *p* > .05^c^	Not possible to calculate OR
Cognitive^d^					
Mickens et al. (2019) United States	Battelle Developmental Inventory Overall sample x̅: 8.99 SD = 4.58 English sample x̅: 9.92 SD = 4.74 Spanish sample x̅: 7.98 SD = 4.17 English TEMA x̅: 86.51 SD = 11.83 Spanish TEMA x̅: 75.94 SD = 12.72	31.3%	N/R	TEMA English Low FS β −0.02 (−3.30, 2.44) TEMA Spanish Low FS β −0.07 (−5.77, 2.15)	TEMA English Low FS OR_a_ 0.98 (0.04–11.47)^e^
Hobbs & King (2018) United States	Peabody Picture Vocabulary Test x̅ = 86.2 Woodcock–Johnson letter–word identification x̅ = 99.9	19.2% HFI 4.0% very low FS	N/R	Peabody vocabulary 10th β −0.01 (−0.20 to 0.19) 25th β −0.11 (−0.26 to 0.05) 50th β −0.16 (−0.29 to −0.02) 75th β −0.19 (−0.33 to −0.04) 90th β −0.14 (−0.24 to −0.04) Woodcock−Johnson letter–word 10th β −0.16 (−0.30 to −0.01) 25th β −0.01 (−0.16 to 0.14) 50th β −0.07 (−0.18 to 0.05) 75th β −0.03 (−0.16 to 0.10) 90th β −0.03 (−0.08 to 0.14)	Peabody vocabulary 10th OR_a_ 0.99 (0.82–1.21)^e^ Woodcock−Johnson letter–word 10th OR_a_ 0.85 (0.74–0.99)^e^
Huang et al. (2018) United States	N/R	11.7% HFI_US 15.4% HFI_IMM	Children HFI_US Math test score x̅ = 24.95 Reading test score x̅ = 21.36 Children HFI_IMM Math test score x̅ = .45 Reading test score x̅ = 19.92	Reading assessment score HFI_US β 0.39 SE (1.23) Math assessment score HFI_US β −0.17 SE (1.11)	Reading assessment score HFI_IMM OR_a_ 0.44 (0.10–1.97)^e^ Math assessment score HFI_IMM OR_a_ 0.47 (0.09–2.38)^e^
Johnson & Markowitz, 2018 UnitedStates	Bayley cognitive score at 9 months x̅ = 76.57 SD = 10.00 Bayley cognitive score at 2 years x̅ = 124.78 SD = 10.12	Low FS at 9 months x̅ =0.15 Very low FS at 9 months x̅ =0.04 Low FS at 2 years x̅ = 0.11 Very low FS at 2 years x̅ = 0.03 Low FS at preschool x̅ =0.16 Very low FS at preschool x̅ =0.05	Bayley cognitive score at 9 months HFI at 9 months x̅ = 76.39 SD = 10.10 HFI at 2 years x̅ = 76.42 SD = 9.90 HFI at preschool x̅ = 76.38 SD = 9.75Bayley cognitive score (2 years) HFI at 9 months x̅ = 124.58 SD = 9.98 HFI at 2 years x̅ = 124.52 SD = 10.32 HFI at preschool x̅ = 124.32 SD = 10.20	Approaches to learning Low FS at 2 years β −0.10 SE (0.08)very low FS at 2 years β −0.34 SE (0.12)Reading Low FS at 2 years β −0.13 SE (0.06)very low FS at 2 years β −0.07 SE (0.11)Math Low FS at 2 years β −0.18 SE (0.07) very low FS at 2 years β −0.27 SE (0.12)	Reading Low FS at 2 years eORa 0.88 (0.78–0.99)Math Low FS at 2 years eORa 0.84 (0.73–0.96)
Obradovíc, Yousafzai, Finch, & Rasheed (2016) Pakistan	N/R	33.0%	N/R	EF β 0.022 SE (0.026)^c^ Verbal IQ β 0.048 SE (0.025)^c^ Performance IQ β 0.042 SE (0.026)^c^	Verbal IQ OR 0.95 (0.91–1.00)^e^
Hernandez & Jacknowitz, (2009) United States	N/R	HIF at 24 months x̅ = 24.54, SEM = 0.09 HIF persistent x̅ = 24.58, SEM = 0.10	Cognitive scores at 24 months HFI at 9 months x̅ = 125.66, SEM = 0.55 HFI at 24 months x̅ = 123.93, SEM = 0.70 HFI persistent x̅ = 123.93, SEM = 0.77	Cognitive scores at 24 months Adult HIF persistent β −0.87 SE (0.81) Adult HFI at 9 months β 0.41 SE (0.54) Adult HFI at 24 months β −1.50 SE (0.69)	
Zaslow et al., (2009) United States High income	Mental development score x̅ = 34.7 SD = 22.4	2.6% very low FS 9.9% low FS	N/R	3−level FI and mental development β = 0.015	Not possible to calculate OR
Motor^d^					
Milner et al., (2018) Kenya	N/R	HFI timing Round 1 x̅ = 0.94 SD = 0.51 Round 2 x̅ = 0.88 SD = 0.46 Round 3 x̅ = 0.81 SD = 0.44 Round 4 x̅ = 0.83 SD = 0.40 Round 5 x̅ = 0.83 SD = 0.43 Round 6 x̅ = 0.80 SD = 0.45 Round 7 x̅ = 0.80 SD = 0.41 Round 8 x̅ = 0.80 SD = 0.44 Round 9 x̅ = 0.84 SD = 0.47 HFI intensity Cumulative 24 months x̅ = 7.54 SD = 2.98 HFI duration Never 35% 1–2 times 33.0% 3–4 times 15.0% 5–6 times 9.0% ≥7 times 7.0%	N/R	Gross motor HFI 3 months ago β −0.097 (−0.23, 0.036) HFI current β −0.050 (−0.18, 0.079) HFI intensity β −0.028 (−0.058, 0.0019) HFI duration β −0.029 (−0.066, 0.0075)	Gross motor HFI 3 months ago OR_a_ 0.91 (0.79–1.03)^e^
Hernandez & Jacknowitz, (2009) United States	N/R	HIF at 24 months x̅ = 24.54, SEM = 0.09 HIF persistent x̅ = 24.58, SEM = 0.10	Motor scores at 24 months HFI at 9 months x̅ = 81.65, SEM = 0.29 HFI at 24 months x̅ = 81.48, SEM = 0.36 HFI persistent x̅ = 80.86, SEM = 0.39	Motor scores at 24 months Adult HIF persistent β −0.58 SE (0.40) Adult HFI at 9 months β 0.10 SE (0.29) Adult HFI at 24 months β 0.05 SE (0.35)	Motor scores at 24 months Adult HFI at 24 months OR_a_ 1.05 (0.53–2.09)^e^
Language^d^					
Milner et al. (2018) Kenya	N/R	HFI timing Round 1 x̅ = 0.94 SD = 0.51 Round 2 x̅ = 0.88 SD = 0.46 Round 3 x̅ = 0.81 SD = 0.44 Round 4 x̅ = 0.83 SD = 0.40 Round 5 x̅ = 0.83 SD = 0.43 Round 6 x̅ = 0.80 SD = 0.45 Round 7 x̅ = 0.80 SD = 0.41 Round 8 x̅ = 0.80 SD = 0.44 Round 9 x̅ = 0.84 SD = 0.47 HFI intensity Cumulative 24 months x̅ = 7.54 SD = 2.98 HFI duration Never 35% 1–2 times 33.0% 3–4 times 15.0% 5–6 times 9.0% ≥7 times 7.0%	N/R	Communication HFI 3 months ago β −0.12 (−0.26, 0.019) HFI current β −0.0070 (−0.14, 0.13) HFI intensity β −0.022 (−0.057, 0.014) HFI duration β −0.020 (−0.063, 0.024)	
Saha et al. (2010) Bangladesh	Language expression M 10.0 IQR (7.0, 15.0) Language comprehension M 37.4 SD (7.80)	N/R	N/R	Language comprehension OR_a_ 0.83 (0.92–0.74)^b^ Language expression OR_a_ 0.36 (0.37–0.36)^b^	

Abbreviations: CI, confidence intervals; CSE, clustered standard errors; ECD, early childhood development; EF, executive function; FS, Food Security; HFI, Household Food Insecurity; IMM, immigrant; IQ, intelligence quotient; IQR, interquartile range; M, median; N/R, not reported; OR, odds ratio; SD, standard deviation; SE: standard error; TEMA: Test of Early Mathematics Ability; US: United States; x̅: mean.

a Percentage of ECD outcome calculated by data provided by the study.

b Effect size calculated per data provided by the study.

c Effect size not adjusted.

d Reported as ECD description adopted in this review.

e Calculated by the authors as recommended by Deeks, Altman, & Bradburn (2001) and Altman (1991).

For the ECD outcomes, the prevalence of developmental risk in the US ranged from 14.0% (Rose‐Jacobs et al., 2008) to 15.2% (Black et al., 2012) among children ages 4 to 36 months and was 11.5% among children ages 4 to 48 months (Drennen et al., 2019). Most studies assessed children's socio‐emotional and cognitive development in the US. Whitaker et al. (2006) evaluated socio‐emotional outcomes in children at 3 years and found high prevalence of inattention/hyperactivity (10.2%), aggressiveness (10.4%), anxiety/depression (12.6%), and overall behavioural disorder (21.9%). Zaslow et al. (2009), investigating children aged 9 and 24 months, found that 38.7% had insecure attachment (i.e., disorganized, avoidant, or ambivalent attachment) (Table 3).

For cognitive outcomes, the studies provided only the average test performance score and can just be interpreted in relation to the minimum and maximum possible range of values or compared with the means of other populations in which the same test is applied. For instance, Zaslow et al. (2009) presented the mean score for mental development from a nationally representative sample of children when they were 24 months old (34.7 ± 22.4, range 0–81.8). Johnson and Markowitz (2018) reported a Bayley cognitive mean score of 124.78 ± 10.12 at 2 years of age (range 40–160). Hobbs and King (2018) found that 5‐year‐old children living in large urban areas of the US had a mean receptive vocabulary score of 86.2 using the Peabody Picture Vocabulary Test (standard 20–160), and the reading mean score was 99.9 using Woodcock–Johnson letter–word identification (standard 100 ± 15).

### 3.3 HFI and ECD associations, meta‐analysis, and sources of heterogeneity

The meta‐analysis was performed for the ECD domains and subdomains identified (for definitions, see Table 1). For developmental risk and cognitive/math and cognitive/school readiness and reading, the studies available were conducted in HICs. Cognitive/vocabulary and motor development studies were conducted in both HICs and LMICs.

**Figure 3:**
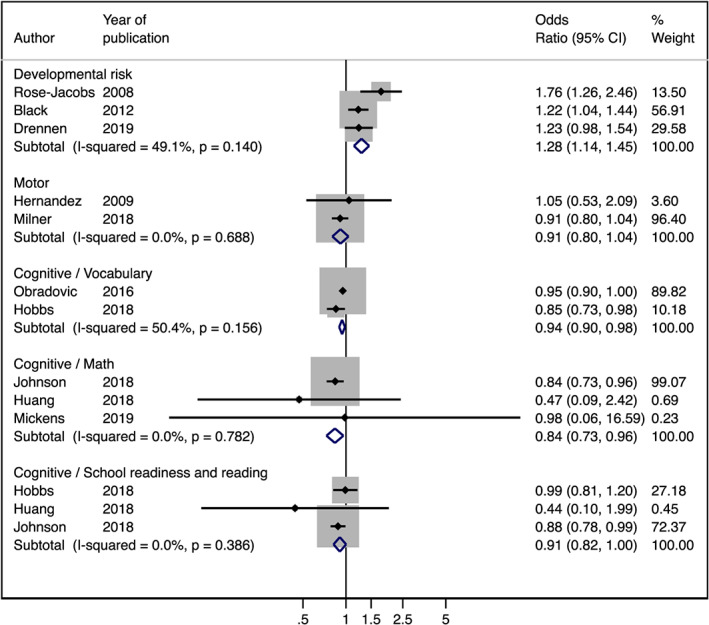
Fixed effects of meta‐analysis of studies evaluating the association between household food insecurity and developmental risk (OR > 1 is considered risk factor) and motor and cognitive outcomes (OR < 1 is considered risk factor)

Fixed‐effects meta‐analysis was performed for developmental risk, motor (fine and gross motor outcomes), cognitive/school readiness and reading, cognitive/vocabulary, and cognitive/math subdomains (Figure 3). HFI was significantly associated with developmental risk (OR 1.28; 95% CI [1.14, 1.45]) and a suboptimal cognitive outcome in math (OR 0.84; 95% CI [0.73, 0.96]) and vocabulary (OR 0.94; 95% CI [0.90, 0.98]) skills. HFI was marginally associated with cognitive/school readiness and reading (OR 0.91; 95% CI [0.82, 1.00]). No association between motor development and HFI was found (0.91 OR; 95% CI [0.80, 1.04]).

**Figure 4:**
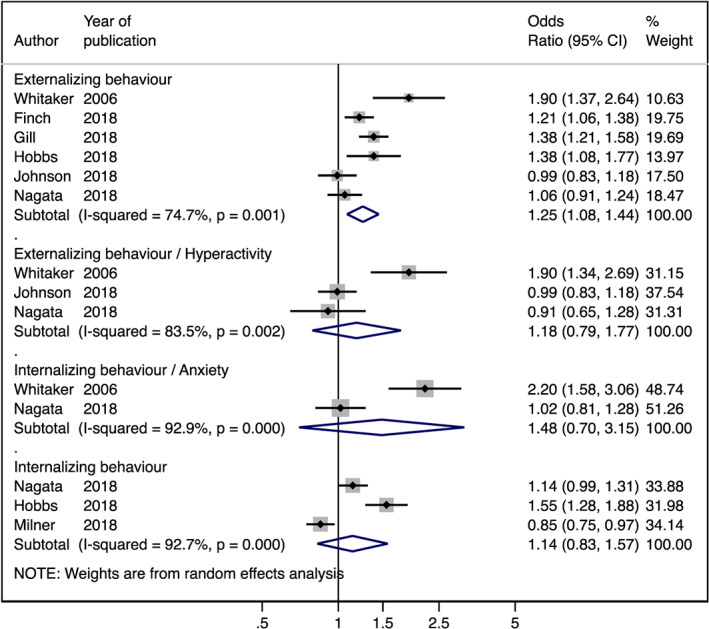
Random effects of meta‐analysis of studies evaluating the association between household food insecurity and socio‐emotional outcomes (OR > 1 is considered risk factor)

Due to the high heterogeneity found among studies, a random‐effects meta‐analysis was performed for socio‐emotional outcomes: externalizing behaviour (OR 1.25; 95% CI [1.08, 1.44]), externalizing behaviour/hyperactivity (OR 1.18; 95% CI [0.79, 1.77]), internalizing behaviour (OR 1.14; 95% CI [0.83, 1.57]), and internalizing behaviour/anxiety (OR 1.48; 95% CI [0.70, 3.15]) (Figure 4). The high heterogeneity identified was possibly due to the lack of standardized (a) sample size, (b) study quality, (c) tools used to measure ECD, and (d) time lapse between assessing ECD and HFI (Table 4).

**Table 4:** Possible sources of heterogeneity by early childhood development socio‐emotional subdomains

		Externalizing behaviour 6 studies^a^ (I‐squared = 74.7%)		Externalizing behaviour/hyperactivity 3 studies^b^ (I‐squared = 83.5%)		Internalizing behaviour 3 studies^c^ (I‐squared = 92.7%)		Internalizing behaviour/anxiety 2 studies^d^ (I‐squared = 92.9%)	
		Number of studies	Possible sources of heterogeneity	Number of studies	Possible sources of heterogeneity	Number of studies	Possible sources of heterogeneity	Number of studies	Possible sources of heterogeneity
Study design	Cross‐sectional	1	Yes	0	No	3	No	0	No
	Longitudinal	5		3		0		2	
Study quality	Strong	3	Yes	2	Yes	0	No	1	Yes
	Moderate	2		1		3		1	
	Weak	1		0		0		0	
Sample size	≤1,000	1	Yes	1	Yes	2	Yes	1	Yes
	1,001–5,000	5		2		1		1	
	5,001–10,000	0		0		0		0	
	≥10,001	0		0		0		0	
Country income	Low–middle	1	Yes	0	No	1	Yes	0	No
	High	5		3		2		2	
Informant	Mother only	4	Yes	2	Yes	3	No	2	No
	Mother and/or caregiver	2		1		0		0	
Size effect	Adjusted	5	Yes	3	No	3	No	2	No
	Crude	1		0		0		0	
HFI intensity	Very low + low	5	Yes	2	Yes	3	No	2	No
	Low	1		1		0		0	
HFI tool	CFSM/HFSSM	5	Yes	3	No	2	Yes	2	No
	HFIAS	1		0		1		0	
ECD tool	PEDS	0	Yes	0	Yes	0	Yes	0	No
	BSF‐R	0		0		0		0	
	ASQ‐1	0		0		1		0	
	CBCL	3		2		2		2	
	Others	3		1		0		0	
HFI and ECD evaluation	At the same time	3	Yes	1	Yes	2	Yes	1	Yes
	≤12 months	2		2		1		1	
	>12 months	1		0		0		0	

Abbreviations: ASQ‐1, Ages and Stages Questionnaire; BSF‐R, Bayley Short Form‐Research Edition; CBCL, Child Behavior Checklist; CFSM, Core Food Security Module; ECD, early childhood development; HFI, household food insecurity; HFIAS, Household Food Insecurity Access Scale; HFSSM, Household Food Security Survey Module; PEDS, Parents Evaluation of Developmental Status.

a Finch, Yousafzai, Rasheed, & Obradović (2018); Gill et al. (2018); Hobbs & King (2018); Johnson & Markowitz (2018); Nagata et al. (2018); and Whitaker et al. (2006).

b Johnson & Markowitz (2018); Nagata, Gomberg, Hagan, Heyman, & Wojcicki (2018); and Whitaker et al. (2006).

c Nagata, Gomberg, Hagan, Heyman, & Wojcicki (2018); Hobbs & King (2018); and Milner, Fiorella, Mattah, Bukusi, & Fernald (2018).

d Nagata, Gomberg, Hagan, Heyman, & Wojcicki (2018) and Whitaker et al. (2006).

The language domain was assessed in different aspects for only two studies conducted in LMICs; thus, no meta‐analysis was performed. Findings on the language domain individual studies showed that communication at 24 months was not associated with HFI (Milner et al., 2018); however, comprehensive and expressive language skills at 18 months old were significantly associated with HFI (Saha et al., 2010).

## 4 DISCUSSION

This systematic review found that HFI is associated with poor ECD outcomes among children under 5 years old in both HICs and LMICs. Meta‐analysis showed that HFI was associated with the developmental risk and cognitive outcomes related to vocabulary and math skills. These results are consistent with previous reviews focusing on HICs (Cook & Frank, 2008; Pérez‐Escamilla & Vianna, 2012; Shankar et al., 2017) and reinforce the critical role that adverse experiences, such as HFI, during early childhood have in putting at risk the future of vulnerable children.

We hypothesize that HFI can impact child cognitive development through several pathways. First, HFI is strongly related with poverty (Wight, Kaushal, Waldfogel, & Garfinkel, 2014), and persistent poverty is known to have negative effects on children's cognitive development (Schoon, Jones, Cheng, & Maughan, 2012). Second, HFI is also associated with a poorer diet quality and quantity (Fram, Ritchie, Rosen, & Frongillo, 2015; Prado & Dewey, 2014), resulting in malnutrition (Miller, Murray, Thomson, & Arbour, 2016), which in turn has been associated with impaired brain development (Aboud & Yousafzai, 2016), leading to cognitive deficits, poor school achievement, and high rates of school dropout (Chilton, Chyatte, & Breaux, 2007; Victora et al., 2003; Walker, Chang, Powell, & Grantham‐McGregor, 2005). Third, food insecurity‐related psycho‐emotional stress may lead to difficulties in concentrating and learning (Pérez‐Escamilla & Vianna, 2012). This can also negatively affect caregivers' mental health status leading to less responsive caregiving and early childhood stimulation opportunities at home (Fuller et al., 2002; Pérez‐Escamilla & Vianna, 2012). Hence, we hypothesize that HFI is associated with poverty, poor diet quality, and nutritional deficiencies as well as expose families to stress and anxiety that impairs parent–child interaction, which may put children at developmental risk.

Despite the high heterogeneity, the meta‐analysis results identified HFI to be a risk factor for externalizing behavioural problems, which is consistent with previous studies (Gill et al., 2018; Hobbs & King, 2018; Johnson & Markowitz, 2018; Whitaker et al., 2006). High levels of poverty‐related stressors, such as HFI (Cook et al., 2008; Pérez‐Escamilla & Vianna, 2012), could lead to behaviour problems which in turn can seriously disrupt caregiver–child interactions (McLeod & Fettes, 2007). The high heterogeneity observed among the studies examining socio‐emotional development limits the strength of our conclusions in this domain.

Our meta‐analysis did not find associations between HFI and motor development, which is consistent with previous studies (Hernandez & Jacknowitz, 2009; Milner et al., 2018). Few studies have investigated these outcomes; hence, these findings should be interpreted with caution. Although recent research indicates that factors such as adult–child interactions and caregiving practices contribute to motor development (Adolph, Vereijken, & Shrout, 2003; Kariger et al., 2005; Kuklina, Ramakrishnan, Stein, Barnhart, & Martorell, 2004), the motor domain seems to be less susceptible to environmental influences than the other domains (Fernald et al., 2017). Both genetic and environmental factors are likely to play a key role, but the interactions between environment and genes are complex and not completely understood (Slining, Adair, Goldman, Borja, & Bentley, 2010).

Language and cognitive domains are highly interdependent, because language‐promoting activities support cognitive development (McClure, Cunningham, Bull, Berman, & Allison, 2018). Only two studies examined the association of HFI with language development, thus this relationship needs to be further investigated. In addition, further prospective studies evaluating HFI and cognitive/school readiness and reading need to be conducted to clarify this association.

Our findings should also be interpreted considering some limitations. Studies used different tools to assess HFI and ECD. This lack of uniformity, especially in the measurement of ECD and its domains, limits the interpretation of the data and must be considered when generalizing our findings. On the other hand, HFI was measured using similar experience‐based HFI scales that have been shown to be reliable globally (Marques et al., 2015; Pérez‐Escamilla et al., 2017). HFI experience‐based scales adapted from the HFSSM have been applied in different socio‐economic and cultural contexts and have demonstrated adequate psychometrics, reliability, and validity of the instrument properties (Marques et al., 2015; Pérez‐Escamilla et al., 2017). Our findings call for the development of standardized tools to measure ECD globally, which will allow for greater comparability between studies. Furthermore, future studies need to clearly report when and how HFI and ECD were measured. Child's age at the time of assessment can be a strong confounder when comparing findings across studies (Johnson & Markowitz, 2018); hence, it must be clearly reported.

Studies with different geographic populations and age groups were included in this review, which should be considered in the interpretation of the results. Although we considered performing a meta‐regression to explore the results according to the country's income, due to the very limited number of studies performed on LMICs, this was not possible. Our review and meta‐analysis clearly demonstrate the need to conduct prospective studies to investigate the associations between HFI and ECD in LMICs. Despite these limitations, our meta‐analysis was carefully carried out using random effects models for domains with high heterogeneity across studies (Deeks et al., 2008; Egger, Davey‐Smith, & Altman , 2008). Another limitation is that the majority of studies were cross‐sectional or presenting cross‐sectional analysis for the purposes this review, which precludes drawing causal inferences (Atkins et al., 2004). The sources of bias identified through this systematic review can help guide the design of future studies (Boutron et al., 2019).

To our knowledge, this is the first review and meta‐analysis that identifies HFI as a risk factor for suboptimal ECD, which includes results from LMICs. Our findings suggest that the associations between HFI and ECD can vary across ECD domains. HFI was associated with developmental risk and poor cognitive outcomes, including vocabulary and math skills, among children under 5 years old. This finding supports the need to consider HFI as a key component of integrated ECD national strategies (Pérez‐Escamilla, 2017).

## ACKNOWLEDGMENT

All those who contributed to the study are already identified as authors. This study was financed in part by the Coordenação de Aperfeiçoamento de Pessoal de Nível Superior (CAPES)—Brasil (Finance Code 001).

## CONFLICTS OF INTEREST

The authors declare that they have no conflicts of interest.

## CONTRIBUTIONS

KHDO, GMA, MBG, ASM, AMS, DCH, RPE, and GB conceptualized and designed the study, reviewed, and revised the manuscript. KHDO and GMA conducted the bibliographic search, collected data, and selected the studies. MBG and GB coordinated and supervised data collection. GMA standardized the data. ASM resolved the disagreements between the first reviewers. AMS reviewed the main study tables. GB carried out the meta‐analysis. KHDO, MBG, GB, and RPE interpreted the results. KHDO, MBG, and GB drafted the initial manuscript. MBG, GB, DCH, and RPE revised the article critically for important intellectual content. KHDO, GMA, MBG, ASM, AMS, DCH, RPE, and GB agree to be accountable for all aspects of the work.

## Supporting information


**Data S1:** Databases and Individualized Truncations of Words

**Data S2:** Excluded studies in the systematic review

**Data S3:** Quality Assessment Tool for Quantitative Studies. Original check‐list and adapted items
