# Supplementary material for: Household food insecurity and early childhood development: Systematic review and meta‐analysis
Source: Matern Child Nutr. 2020 Feb 12;16(3):e12967. doi: 10.1111/mcn.12967 (PMC7296813; doi:10.1111/mcn.12967)
Supplement: Supplementary file 1 — Data S1: Databases and Individualized Truncations of Words [file MCN-16-e12967-s001.docx]

**Appendix 1** Databases and Individualized Truncations of Words

| Database | Descriptors used |
| --- | --- |
| PudMed | ("Infant, Newborn” OR Neonate OR Child OR “Child, Preschool” OR Toddler) AND “Food Insecurity” AND (“Food and Nutrition Security” OR “Food Supply” OR "Food Security") AND (“Child Development” OR “Infant Development” OR “Toddler Development” OR “Developmental Disabilities” OR “Child Development Deviations” OR “Child Development Disorders” OR “Child Development Disorders, Specific” OR “Developmental Delay Disorders” OR “Disabilities, Developmental” OR Development OR “Postnatal Development” OR Cognitive OR Motor OR Psychosocial OR "Emotional Development" OR "Child Behaviour" OR Language OR Speech OR "Receptive Language" OR "Child Responsiveness" OR "Child Compliance" OR “Neurodevelopmental Disorders” OR “Physical Development”) |
| LILACS | ( ( ( ( ( ( ( ( "LACTENTE" ) or "RECEM--NASCIDO" or "RECEM--NASCIDOS" or "RECEM-DESMAMADOS.." ) or "CRIANCA" or "CRIANCA POS-TERMO" or "CRIANCA PRE-ESCOLAR" ) or "CRIANCA RECEM-NASCIDA" ) or "NEONATO" or "NEONATO PRE-TERMO" ) or "NINO" or "NINO PREESCOLAR" ) or "RECIEN-NACIDO" or "RECIEN-NACIDOS" ) or "PREESCOLAR" or "PREESCOLARES" ) or "INFANTE" or "INFANTES" or "INFANTES/NINOS" or "INFANTIL" or "INFANTIL-CHILD" or "INFANTIL-NINOS" [Palavras] and ( ( ( ( "SEGURANCA ALIMENTAR" or "SEGURANCA ALIMENTAR E NUTRICIONAL" ) or "ABASTECIMENTO DE ALIMENTOS" or "ABASTECIMIENTO DE ALIMENTOS" ) or "SEGURIDAD ALIMENTARIA" or "SEGURIDAD ALIMENTARIA Y NUTRICIONAL" ) or "INSEGURANCAALIMENTAR" or "INSEGURIDADALIMENTARIA" ) [Palavras] and ( ( ( ( ( ( ( ( ( ( ( ( ( ( ( ( ( ( ( ( ( ( "DESENVOLVIMENTO INFANTIL" or "DESENVOLVIMENTO PRE-ESCOLAR" ) or "DESENVOLVIMENTO" or "DESENVOLVIMENTO DA CRIANCA" or "DESENVOLVIMENTO DA CRIANCA EM IDADE PRE-ESCOLAR" or "DESENVOLVIMENTO DA CRIANCA PRE-ESCOLAR" or "DESENVOLVIMENTO DA LINGUAGEM" or "DESENVOLVIMENTO DA PERSONALIDADE" or "DESENVOLVIMENTO DAS CRIANCAS" or "DESENVOLVIMENTO DAS CRIANCAS EM IDADE PRE-ESCOLAR" ) or "DEFICIENCIAS DO DESENVOLVIMENTO" ) or "DESVIOS DO DESENVOLVIMENTO INFANTIL" ) or "TRANSTORNOS DO DESENVOLVIMENTO DA LINGUAGEM" or "TRANSTORNOS DO DESENVOLVIMENTO INFANTIL" ) or "TRANSTORNOS DO NEURODESENVOLVIMENTO" ) or "TRANSTORNOS ESPECIFICOS DO DESENVOLVIMENTO INFANTIL" ) or "COGNITIVO" or "COGNITIVO-COMPORTAMEMTAL" or "COGNITIVO-COMPORTAMENTAIS" or "COGNITIVO-EMOCIONAIS" or "COGNITIVO-EMOCIONAL" or "COGNITIVO-MOTOR" or "COGNITIVO-MOTORA" or "COGNITIVO-SOCIAIS" or "COGNITIVO-SOCIALES" ) or "MOTOR" ) or "PSICOSSOCIAL" ) or "COMPORTAMENTO INFANTIL" ) or "LINGUAGEM" or "LINGUAGEM INFANTIL" or "LINGUAGEM-COGNICAO" ) or "FALA" or "FALA-LINGUAGEM" or "FALA/LINGUAGEM" or "FALA/MOTRICIDADE" ) or "NEURODESENVOLVIMENTO" or "NEURODEVELOPMENTAL DISORDER" ) or "DESARROLLO" or "DESARROLLO DE LACTANTES" or "DESARROLLO DE LOS LACTANTES" or "DESARROLLO DE LOS NINOS" or "DESARROLLO DE LOS NINOS EN EDAD PREESCOLAR" or "DESARROLLO DE LOS PREESCOLARES" ) or "DISCAPACIDADES DEL DESARROLLO" ) or "TRANSTORNOS ESPECIFICOS DO DESENVOLVIMENTO INFANTIL" ) or "COGNICION" or "COGNITION DISORDERS" ) or "MOTORA" or "MOTORA-COGNITIVA" or "MOTORA-COGNITIVAS" or "MOTORAS/FISICAS" ) or "DESARROLLO INFANTIL" or "CONDUCTA INFANTIL" ) or "LENGUAJE" or "LENGUAJE DEL NINO" or "LENGUAJE INFANTIL" or "LENGUAJE/APRENDIZAJE" ) or "HABLA" or "HABLA-LENGUAJE" or "HABLA/MOTRICIDAD" ) [Palavras] |
| SCOPUS | ( TITLE-ABS-KEY ( "Infant, Newborn" OR neonate OR child OR "Child, Preschool" OR toddler ) ) AND ( ALL ( "Food Insecurity" ) ) AND ( TITLE-ABS-KEY ( "Food and Nutrition Security" OR "Food Supply" OR "Food Security" ) ) AND ( ALL ( "Child Development" OR "Infant Development" OR "Toddler Development" OR "Developmental Disabilities" OR "Child Development Deviations" OR "Child Development Disorders" OR "Child Development Disorders, Specific" OR "Developmental Delay Disorders" OR "Disabilities, Developmental" OR development OR "Postnatal Development" OR cognitive OR motor OR psychosocial OR "Emotional Development" OR "Child Behaviour" OR language OR speech OR "Receptive Language" OR "Child Responsiveness" OR "Child Compliance" OR "Neurodevelopmental Disorders" OR "Physical Development" ) ) |
| Web of Science | TS=("Infant, Newborn” OR Neonate OR Child OR “Child, Preschool” OR Toddler) AND TS=(“Food Insecurity”) AND TS=(“Food and Nutrition Security” OR “Food Supply” OR "Food Security") AND TS=(“Child Development” OR “Infant Development” OR “Toddler Development” OR “Developmental Disabilities” OR “Child Development Deviations” OR “Child Development Disorders” OR “Child Development Disorders, Specific” OR “Developmental Delay Disorders” OR “Disabilities, Developmental” OR Development OR “Postnatal Development” OR Cognitive OR Motor OR Psychosocial OR "Emotional Development" OR "Child Behaviour" OR Language OR Speech OR "Receptive Language" OR "Child Responsiveness" OR "Child Compliance" OR “Neurodevelopmental Disorders” OR “Physical Development”) |
| PsycInfo | Any Field: ("Infant, Newborn” OR Neonate OR Child OR “Child, Preschool” OR Toddler) AND Any Field: (“Food Insecurity”) AND Any Field: (“Food and Nutrition Security” OR “Food Supply” OR "Food Security") AND Any Field: (“Child Development” OR “Infant Development” OR “Toddler Development” OR “Developmental Disabilities” OR “Child Development Deviations” OR “Child Development Disorders” OR “Child Development Disorders, Specific” OR “Developmental Delay Disorders” OR “Disabilities, Developmental” OR Development OR “Postnatal Development” OR Cognitive OR Motor OR Psychosocial OR "Emotional Development" OR "Child Behaviour" OR Language OR Speech OR "Receptive Language" OR "Child Responsiveness" OR "Child Compliance" OR “Neurodevelopmental Disorders” OR “Physical Development”) |
| Science Direct | All (Infant, Newborn OR Neonate OR Child OR Child, Preschool OR Toddler) AND All (Food Insecurity) AND All (Food and Nutrition Security OR Food Supply OR Food Security) AND All (Child Development OR Infant Development OR Toddler Development OR Developmental Disabilities OR Child Development Deviations OR Child Development Disorders OR Child Development Disorders, Specific OR Developmental Delay Disorders OR Disabilities, Developmental OR Development OR Postnatal Development OR Cognitive OR Motor OR Psychosocial OR Emotional Development OR Child Behaviour OR Language OR Speech OR Receptive Language OR Child Responsiveness OR Child Compliance OR Neurodevelopmental Disorders OR Physical Development) |
| Embase | ('infant, newborn' OR neonate OR child OR 'child, preschool' OR toddler) AND 'food insecurity' AND ('food and nutrition security' OR 'food supply' OR 'food security') AND ('child development' OR 'infant development' OR 'toddler development' OR 'developmental disabilities' OR 'child development deviations' OR 'child development disorders' OR 'child development disorders, specific' OR 'developmental delay disorders' OR 'disabilities, developmental' OR development OR 'postnatal development' OR cognitive OR motor OR psychosocial OR 'emotional development' OR 'child behaviour' OR language OR speech OR 'receptive language' OR 'child responsiveness' OR 'child compliance' OR 'neurodevelopmental disorders' OR 'physical development') |
| Google Scholar | ("Infant, Newborn” OR Neonate OR Child OR “Child, Preschool” OR Toddler) AND “Food Insecurity” AND (“Food and Nutrition Security” OR “Food Supply” OR "Food Security") AND (“Child Development” OR “Infant Development” OR “Toddler Development” OR “Developmental Disabilities” OR “Child Development Deviations” OR “Child Development Disorders” OR “Child Development Disorders, Specific” OR “Developmental Delay Disorders” OR “Disabilities, Developmental” OR Development OR “Postnatal Development” OR Cognitive OR Motor OR Psychosocial OR "Emotional Development" OR "Child Behaviour" OR Language OR Speech OR "Receptive Language" OR "Child Responsiveness" OR "Child Compliance" OR “Neurodevelopmental Disorders” OR “Physical Development”) |
| Open Grey | ABSTRACT:("Infant, Newborn” OR Neonate OR Child OR “Child, Preschool” OR Toddler) AND ABSTRACT:“Food Insecurity” AND ABSTRACT:(“Food and Nutrition Security” OR “Food Supply” OR "Food Security") AND ABSTRACT:(“Child Development” OR “Infant Development” OR “Toddler Development” OR “Developmental Disabilities” OR “Child Development Deviations” OR “Child Development Disorders” OR “Child Development Disorders, Specific” OR “Developmental Delay Disorders” OR “Disabilities, Developmental” OR Development OR “Postnatal Development” OR Cognitive OR Motor OR Psychosocial OR "Emotional Development" OR "Child Behaviour" OR Language OR Speech OR "Receptive Language" OR "Child Responsiveness" OR "Child Compliance" OR “Neurodevelopmental Disorders” OR “Physical Development”) |
|  |  |
