# Supplementary material for: Household food insecurity and early childhood development: Systematic review and meta‐analysis
Source: Matern Child Nutr. 2020 Feb 12;16(3):e12967. doi: 10.1111/mcn.12967 (PMC7296813; doi:10.1111/mcn.12967)
Supplement: Supplementary file 2 — Data S2: Excluded studies in the systematic review [file MCN-16-e12967-s002.docx]

**Appendix 2** Excluded studies in the systematic review

| **Reasons for exclusion** | **References** |
| --- | --- |
| Review papers, conference abstracts, study protocol | (DeBoer et al., 2018; Kaiser & Townsend, 2005; Melchior et al., 2014; Symington et al., 2018; Weitzman et al., 2013) |
| Age over 5 years | (Belsky, Moffitt, Arseneault, Melchior, & Caspi, 2010; Casey et al., 2005; Frongillo, Jyoti, & Jones, 2006; Gee, 2018; Howard, 2011; Huang, Oshima, & Kim, 2010; Huang & Vaughn, 2016; Jyoti, Frongillo, & Jones, 2005; Kimbro & Denney, 2015; Melchior et al., 2009; Melchior et al., 2012; Parchment, Small, Osuji, McKay, & Bhana, 2016; Ramsey, Giskes, Turrell, & Gallegos, 2011; Slopen, Fitzmaurice, Williams, & Gilman, 2010; Weinreb et al., 2002; Winicki & Jemison, 2003) |
| Does not assess association between Household Food Insecurity and Early Childhood Development | (Anjum, Asim, Awan, Saghir, & Nawaz, 2016; Castro et al., 2017; Cook et al., 2008; D. B. Cutts et al., 2011; Frank et al., 2010; Gorman, Zearley, & Favasuli, 2011; Horm et al., 2018; Jeharsae, Sangthong, Wichaidit, & Chongsuvivatwong, 2013; Johnson & Markowitz, 2018; King, 2016; Knowles, Rabinowich, Ettinger de Cuba, Cutts, & Chilton, 2016; Ryu & Bartfeld, 2012; Slack & Yoo, 2005; Vazir et al., 2013; Zilanawala & Pilkauskas, 2012) |
| Repeated sample | (Diana Becker Cutts et al., 2011; Milner, 2016) |

Anjum, F., Asim, M., Awan, K. A., Saghir, A., & Nawaz, Y. (2016). Household food insecurity and its impact on children health in rural faisalabad pakistan. *Rawal Medical Journal, 41*, 373-376.

Belsky, D. W., Moffitt, T. E., Arseneault, L., Melchior, M., & Caspi, A. (2010). Context and sequelae of food insecurity in children's development. *Am J Epidemiol, 172*, 809-818.

Casey, P. H., Szeto, K. L., Robbins, J. M., Stuff, J. E., Connell, C., Gossett, J. M., & Simpson, P. M. (2005). Child health-related quality of life and household food security. *Arch Pediatr Adolesc Med, 159*(1), 51-56.

Castro, F., Place, J. M., Villalobos, A., Rojas, R., Barrientos, T., & Frongillo, E. A. (2017). Poor early childhood outcomes attributable to maternal depression in mexican women. Arch Womens Ment Health, 20, 561-568.

Cook, J. T., Frank, D. A., Casey, P. H., Rose-Jacobs, R., Black, M. M., Chilton, M., . . . Cutts, D. B. (2008). A brief indicator of household energy security: Associations with food security, child health, and child development in us infants and toddlers. *Pediatrics, 122*, e867-e875.

Cutts, D. B., Meyers, A. F., Black, M. M., Casey, P. H., Chilton, M., Cook, J. T., . . . Coleman, S. (2011). Us housing insecurity and the health of very young children. *American Journal of Public Health, 101*, 1508-1514.

Cutts, D. B., Meyers, A. F., Black, M. M., Casey, P. H., Chilton, M., Cook, J. T., . . . Frank, D. A. (2011). Us housing insecurity and the health of very young children. *Am J Public Health, 101*, 1508-1514.

DeBoer, M. D., Platts-Mills, J. A., Scharf, R. J., McDermid, J. M., Wanjuhi, A. W., Gratz, J., . . . Mduma, E. (2018). Early life interventions for childhood growth and development in tanzania (elicit): A protocol for a randomised factorial, double-blind, placebo-controlled trial of azithromycin, nitazoxanide and nicotinamide. *BMJ Open, 8*, e021817.

Frank, D. A., Casey, P. H., Black, M. M., Rose-Jacobs, R., Chilton, M., Cutts, D., . . . Cook, J. T. (2010). Cumulative hardship and wellness of low-income, young children: Multisite surveillance study. *Pediatrics, 125*, e1115-e1123.

Frongillo, E. A., Jyoti, D. F., & Jones, S. J. (2006). Food stamp program participation is associated with better academic learning among school children. *Journal of Nutrition, 136*, 1077-1080.

Gee, K. A. (2018). Growing up with a food insecure adult: The cognitive consequences of recurrent versus transitory food insecurity across the early elementary years. *Journal of Family Issues*, 39, 2437–2460.

Gorman, K. S., Zearley, K. K., & Favasuli, S. (2011). Does acculturation matter?: Food insecurity and child problem behavior among low-income, working hispanic households. *Hispanic Journal of Behavioral Sciences, 33*, 152-169.

Horm, D. M., File, N., Bryant, D., Burchinal, M., Raikes, H., Forestieri, N., . . . Cobo-Lewis, A. (2018). Associations between continuity of care in infant-toddler classrooms and child outcomes. *Early Childhood Research Quarterly, 42*, 105-118.

Howard, L. L. (2011). Does food insecurity at home affect non-cognitive performance at school? A longitudinal analysis of elementary student classroom behavior. *Economics of Education Review, 30*(1), 157-176.

Huang, J., Oshima, K. M. M., & Kim, Y. (2010). Does food insecurity affect parental characteristics and child behavior? Testing mediation effects. *Soc Serv Rev, 84*, 381-401.

Huang, J., & Vaughn, M. G. (2016). Household food insecurity and children's behaviour problems: New evidence from a trajectories-based study. *Br J Soc Work, 46*, 993-1008.

Jeharsae, R., Sangthong, R., Wichaidit, W., & Chongsuvivatwong, V. (2013). Growth and development of children aged 1-5 years in low-intensity armed conflict areas in southern thailand: A community-based survey. *Conflict and Health, 7*(1), 1-8.

Johnson, A. D., & Markowitz, A. J. (2018). Food insecurity and family well-being outcomes among households with young children. *Journal of Pediatrics, 196*, 275-282.

Jyoti, D. F., Frongillo, E. A., & Jones, S. J. (2005). Food insecurity affects school children’s academic performance, weight gain, and social skills. *The Journal of nutrition, 135*, 2831-2839.

Kaiser, L. L., & Townsend, M. S. (2005). Food insecurity among us children. *Topics in Clinical Nutrition, 20*, 313-320.

Kimbro, R. T., & Denney, J. T. (2015). Transitions into food insecurity associated with behavioral problems and worse overall health among children. *Health Affairs, 34*, 1949-1955.

King, C. (2016). Soft drinks consumption and child behaviour problems: The role of food insecurity and sleep patterns. *Public Health Nutrition, 20*, 266-273.

Knowles, M., Rabinowich, J., Ettinger de Cuba, S., Cutts, D. B., & Chilton, M. (2016). "Do you wanna breathe or eat?": Parent perspectives on child health consequences of food insecurity, trade-offs, and toxic stress. *Maternal and child health journal, 20*(1), 25-32.

Melchior, M., Caspi, A., Howard, L. M., Ambler, A. P., Bolton, H., Mountain, N., & Moffitt, T. E. (2009). Mental health context of food insecurity: A representative cohort of families with young children. *Pediatrics, 124*, e564-572.

Melchior, M., Chastang, J. F., Falissard, B., Galèra, C., Tremblay, R., Côtè, S., & Boivin, M. (2014). Epa-0669 – food insecurity and children's symptoms of hyperactivity and inattention. *European Psychiatry, 29*, 1.

Melchior, M., Chastang, J. F., Falissard, B., Galéra, C., Tremblay, R. E., Côté, S. M., & Boivin, M. (2012). Food insecurity and children's mental health: A prospective birth cohort study. *PLoS ONE, 7*, e52615.

Milner, E. M. (2016). *Environmental determinants of early childhood development in rural kenya.* (78), ProQuest Information & Learning, US.

Parchment, T. M., Small, L., Osuji, H., McKay, M., & Bhana, A. (2016). Familial and contextual influences on children’s prosocial behavior: South african caregivers as adult protective shields in enhancing child mental health. *Glob Soc Welf, 3*(1), 1-10.

Ramsey, R., Giskes, K., Turrell, G., & Gallegos, D. (2011). Food insecurity among australian children: Potential determinants, health and developmental consequences. *Journal of Child Health Care, 15*, 401-416.

Ryu, J. H., & Bartfeld, J. S. (2012). Household food insecurity during childhood and subsequent health status: The early childhood longitudinal study—kindergarten cohort. *Am J Public Health, 102*, e50-55.

Slack, K. S., & Yoo, J. (2005). Food hardship and child behavior problems among low-income children. *Social Service Review, 79*, 511-536.

Slopen, N., Fitzmaurice, G., Williams, D. R., & Gilman, S. E. (2010). Poverty, food insecurity, and the behavior for childhood internalizing and externalizing disorders. *Journal of the American Academy of Child and Adolescent Psychiatry, 49*, 444-452.

Symington, E. A., Baumgartner, J., Malan, L., Zandberg, L., Ricci, C., & Smuts, C. M. (2018). Nutrition during pregnancy and early development (nuped) in urban south africa: A study protocol for a prospective cohort. *BMC Pregnancy Childbirth, 18*(1), 308.

Vazir, S., Engle, P., Balakrishna, N., Griffiths, P. L., Johnson, S. L., Creed‐Kanashiro, H., . . . Bentley, M. E. (2013). Cluster‐randomized trial on complementary and responsive feeding education to caregivers found improved dietary intake, growth and development among rural indian toddlers. *Maternal & child nutrition, 9*(1), 99-117.

Weinreb, L., Wehler, C., Perloff, J., Scott, R., Hosmer, D., Sagor, L., & Gundersen, C. (2002). Hunger: Its impact on children’s health and mental health. *Pediatrics, 110*, e41.

Weitzman, M., Baten, A., Rosenthal, D. G., Hoshino, R., Tohn, E., & Jacobs, D. E. (2013). Housing and child health. *Current Problems in Pediatric and Adolescent Health Care, 43*, 187-224.

Winicki, J., & Jemison, K. (2003). Food insecurity and hunger in the kindergarten classroom: Its effect on learning and growth. *Contemporary Economic Policy, 21*, 145-157.

Zilanawala, A., & Pilkauskas, N. V. (2012). Material hardship and child socioemotional behaviors: Differences by types of hardship, timing, and duration. *Child Youth Serv Rev, 34*, 814-825.
