# Supplementary material for: Household food insecurity and early childhood development: Systematic review and meta‐analysis
Source: Matern Child Nutr. 2020 Feb 12;16(3):e12967. doi: 10.1111/mcn.12967 (PMC7296813; doi:10.1111/mcn.12967)
Supplement: Supplementary file 3 — Data S3: Quality Assessment Tool for Quantitative Studies. Original check‐list and adapted items [file MCN-16-e12967-s003.docx]

**Appendix 3** Quality Assessment Tool for Quantitative Studies. Original check-list and adapted items

| **"Effective Public Health Project Practice Quality Assessment Tool" checklist** | |
| --- | --- |
| **Component Ratings** | |
| **Original version** | **Adapted items are underlined** |
| **A)  SELECTION BIAS**  **(Q1) Are the individuals selected to participate in the study likely to be representative of the target population?**  1  Very likely  2  Somewhat likely  3  Not likely  4  Can’t tell  **(Q2) What percentage of selected individuals agreed to participate?**  1  80­–100% agreement  2  60–79% agreement  3  less than 60% agreement  4  Not applicable  5  Can’t tell |  |
| **B)  STUDY DESIGN**  **Indicate the study design**  1  Randomized controlled trial  2  Controlled clinical trial  3  Cohort analytic (two group pre + post)  4  Case-control  5  Cohort (one group pre + post (before and after)  6  Interrupted time series  7  Other specify ______________________  8  Can’t tell  **Was the study described as randomized? If NO, go to Component C.**  No Yes  **If Yes, was the method of randomization described? (See dictionary)**  No Yes  **If Yes, was the method appropriate? (See dictionary)**  No Yes |  |
| **C) CONFOUNDERS**  **(Q1) Were there important differences between groups prior to the intervention?**  1  Yes  2  No  3  Can’t tell  **The following are examples of confounders:**  1  Race  2  Sex  3  Marital status/family  4  Age  5  SES (income or class)  6  Education  7  Health status  8  Pre-intervention score on outcome measure  **(Q2) If yes, indicate the percentage of relevant confounders that were controlled (either in the design (e.g. stratification, matching) or analysis)?**  1  80–100% (most)  2  60–79% (some)  3  Less than 60% (few or none)  4  Can’t Tell | **C) CONFOUNDERS**  **(Q1) Were there important differences between Household Food Insecurity groups vs. Household Food Security prior ECD assessment?**  1  Yes  2  No  3  Can’t tell  **The following are examples of confounders:**  1  Race  2  Sex  3  Marital status/family  4  Age  5  SES (income or class)  6  Education  7  Health status  8  Pre-intervention score on outcome measure  **(Q2) If yes, indicate the percentage of relevant confounders that were controlled (either in the design (e.g. stratification, matching) or analysis)?**  1  80–100% (most)  2  60–79% (some)  3  Less than 60% (few or none)  4  Can’t Tell |
| **D)  BLINDING**  **(Q1) Was (were) the outcome assessor(s) aware of the intervention or exposure status of participants?**  1  Yes  2  No  3  Can’t tell  **(Q2) Were the study participants aware of the research question?**  1  Yes  2  No  3  Can’t tell | **D)  BLINDING**  **(Q1) Was (were) the outcome assessor(s) aware of the HFI status of participants?**  1  Yes  2  No  3  Can’t tell  **(Q2) Were the study participants aware of the research question?**  1  Yes  2  No  3  Can’t tell |
| **E)  DATA COLLECTION METHODS**  **(Q1) Were data collection tools shown to be valid?**  1  Yes  2  No  3  Can’t tell  **(Q2) Were data collection tools shown to be reliable?**  1  Yes  2  No  3  Can’t tell |  |
| **F) WITHDRAWALS AND DROP-OUTS**  **(Q1) Were withdrawals and drop-outs reported in terms of numbers and/or reasons per group?**  1  Yes  2  No  3  Can’t tell  4  Not Applicable (i.e. one time surveys or interviews)  **(Q2) Indicate the percentage of participants completing the study. (If the percentage differs by groups, record the lowest).**  1  80­–100%  2  60–79%  3  less than 60%  4  Can’t tell  5  Not Applicable (i.e. Retrospective case-control) | **F) WITHDRAWALS AND DROP-OUTS**  **(Q1) Were losses in the follow-up – when applicable–or missing data reported in terms of numbers and/or reasons?**  1  Yes  2  No  3  Can’t tell  4  Not Applicable (i.e. one time surveys or interviews)  **(Q2) Indicate the percentage of participants completing the study. (If the percentage differs by groups, record the lowest).**  1  80–100%  2  60–79%  3  less than 60%  4  Can’t tell  5  Not Applicable (i.e. Retrospective case-control) |

Road map for rating quality attributes:

A) SELECTION BIAS

Strong: (Q1 is 1) and (Q2 is 1).

Moderate: (Q1 is 1 or 2) and (Q2 is 2); OR (Q1 is 1 or 2) and (Q2 is 5).

Weak: (Q1 is 3) OR (Q2 is 3); OR (Q1 is 4) and (Q2 is 5).

B) DESIGN

Strong: will be assigned to those articles that described RCTs and CCTs.

Moderate: will be assigned to those that described a cohort analytic study, a case control study, a cohort design, or an interrupted time series.

Weak: will be assigned to those that used any other method or did not state the method used.

C) CONFOUNDERS

Strong: (Q1 is 2) OR (Q2 is 1).

Moderate: (Q1 is 1) and (Q2 is 2).

Weak: (Q1 is 1) and (Q2 is 3); OR (Q1 is 3) and (Q2 is 4).

D) BLINDING

Strong: (Q1 is 2) and (Q2 is 2).

Moderate: (Q1 is 2) OR (Q2 is 2); OR (Q1 is 3) and (Q2 is 3).

Weak: (Q1 is 1) and (Q2 is 1).

E) DATA COLLECTION METHODS

Strong: (Q1 is 1) and (Q2 is 1).

Moderate: (Q1 is 1) and (Q2 is 2) OR (Q2 is 3).

Weak: (Q1 is 2) OR (Q1 is 3 and Q2 is 3).

F) WITHDRAWALS AND DROP-OUTS - a rating of:

Strong: (Q2 is 1).

Moderate: (Q2 is 2) OR (Q2 is 5).

Weak: (Q2 is 3) OR (Q2 is 4).
